# Clinical Prediction of Heart Failure in Hemodialysis Patients: Based on the Extreme Gradient Boosting Method

**DOI:** 10.3389/fgene.2022.889378

**Published:** 2022-04-26

**Authors:** Yanfeng Wang, Xisha Miao, Gang Xiao, Chun Huang, Junwei Sun, Ying Wang, Panlong Li, Xu You

**Affiliations:** ^1^ The School of Electrical and Information Engineering, Zhengzhou University of Light Industry, Zhengzhou, China; ^2^ Department of Clinical Laboratory, The Third Affiliated Hospital, Southern Medical University, Guangzhou, China; ^3^ Department of Medical Statistics, School of Public Health, Sun Yat-sen University, Guangzhou, China

**Keywords:** machine learning, extreme gradient boosting, heart failure prediction, hemodialysis, risk factors

## Abstract

**Background:** Heart failure (HF) is the main cause of mortality in hemodialysis (HD) patients. However, it is still a challenge for the prediction of HF in HD patients. Therefore, we aimed to establish and validate a prediction model to predict HF events in HD patients.

**Methods:** A total of 355 maintenance HD patients from two hospitals were included in this retrospective study. A total of 21 variables, including traditional demographic characteristics, medical history, and blood biochemical indicators, were used. Two classification models were established based on the extreme gradient boosting (XGBoost) algorithm and traditional linear logistic regression. The performance of the two models was evaluated based on calibration curves and area under the receiver operating characteristic curves (AUCs). Feature importance and SHapley Additive exPlanation (SHAP) were used to recognize risk factors from the variables. The Kaplan–Meier curve of each risk factor was constructed and compared with the log-rank test.

**Results:** Compared with the traditional linear logistic regression, the XGBoost model had better performance in accuracy (78.5 vs. 74.8%), sensitivity (79.6 vs. 75.6%), specificity (78.1 vs. 74.4%), and AUC (0.814 vs. 0.722). The feature importance and SHAP value of XGBoost indicated that age, hypertension, platelet count (PLT), C-reactive protein (CRP), and white blood cell count (WBC) were risk factors of HF. These results were further confirmed by Kaplan–Meier curves.

**Conclusions:** The HF prediction model based on XGBoost had a satisfactory performance in predicting HF events, which could prove to be a useful tool for the early prediction of HF in HD.

## Introduction

Heart failure (HF) as a clinical syndrome is one of the main causes of mortality (Ebong et al., 2014; Virani et al., 2021). More than 60 million people are affected by HF worldwide, and the number of HF patients is increasing every year (Groenewegen et al., 2020). Compared with the general population, the prevalence of HF in patients with chronic kidney disease (CKD) is much higher, especially in patients with end-stage renal disease (ESRD) (Rangaswami and McCullough, 2018; Rapa et al., 2019). Currently, hemodialysis (HD) as a renal replacement therapy (RRT) is the main treatment of ESRD (Akash et al., 2014). However, more than 40% of HD patients suffer from HF, which increase the medical care, economic burden, and mortality (House et al., 2019; Sun et al., 2019). Therefore, identification, pre-estimation, and timely interventions can improve the prognosis and prolong the survival time for the population who show a high risk of HF (Abdo, 2017).

The causes of HF in HD patients are multifactorial (Wang and Sanderson, 2011). Similar to the general population, traditional risk factors such as aging, hypertension, diabetes mellitus (DM), and atherosclerosis are associated with HF in HD patients (Liu et al., 2014; Cozzolino et al., 2018). However, excess HF prevalence and mortality in HD population are not fully accounted for by the traditional risk factors (Schefold et al., 2016). Some researchers have found that dialysis can increase the risk of HF events in ESRD patients (Rangaswami and McCullough, 2018; Samanta et al., 2019). Evidence shows that the malnutrition–inflammation syndrome in HD patients is associated with HF (Almeida et al., 2008; Chang et al., 2020). In addition, changed hemodynamic and blood pressure by HD can increase the risk of HF (Dorairajan et al., 2010). Several traditional risk prediction models were proposed to predict the risk of HF in population without HD (Ouwerkerk et al., 2014; Voors et al., 2017). However, these linear models missed specific related factors for HD people and oversimplified the complicated relationships between factors, which may lead to a decrease in performance and miss important risk factors (Mpanya et al., 2021).

Recent advances in extreme gradient boosting (XGBoost), a new integrated machine learning algorithm, have provided a robust method to identify the complex non-linear relationship between multiple variables and outcomes (Kagiyama et al., 2019). This algorithm has strong model generalization ability, fast operating speed, and high model accuracy, which has been applied in orthopedic auxiliary classification, prediction of interaction, and analysis of hypertension-related symptoms to improve accuracy in complex clinical decision-making (Chang et al., 2019; Li and Zhang, 2020; Yu et al., 2020).

Therefore, we aimed to establish a prediction model that integrated patient-specific information and non-traditional factors of HF in HD patients based on XGBoost 1) to accurately predict HF events and 2) to assess the risk of HF in patients with HD treatment.

## Materials and Methods

### Study Population

This retrospective study analyzed the data of 410 ESRD patients who underwent HD treatment in the HD centers of the Third Affiliated Hospital of Southern Medical University and the Third Affiliated Hospital of Sun Yat-sen University between January 2015 and September 2019. All the patients were older than 18 years and received HD treatment for at least 3 months. Patients with the following conditions were excluded from this study: 1) history of renal transplantation; 2) malignancy, acute infection, hepatic, and pulmonary dysfunction; and 3) lack of data on demographic characteristics, laboratory examinations, or physical examination. This study was approved by the Ethics Committee of the Third Affiliated Hospital of Southern Medical University.

### Data Collection

A total of 21 factors were collected at the start of HD therapy. The basic information consisted of the patient’s gender, age, and history of various diseases. After fasting overnight for at least 8 h, the patient’s venous blood samples were collected before dialysis therapy. The laboratory indicators included C-reactive protein (CRP), blood urea nitrogen (BUN), calcium (CA), hemoglobin (HB), phosphorus (P), cholesterol (CHOL), serum creatinine (CRE), lymphocyte (LYMPH), uric acid (UA), low-density lipoprotein cholesterol (LDL-C), high-density lipoprotein cholesterol (HDL-C), intact parathyroid hormone (IPTH), white blood cell count (WBC), platelet count (PLT), and neutrophil count (NEUT). All the abovementioned examinations were conducted at the laboratory centers in the Third Affiliated Hospital of Southern Medical University and the Third Affiliated Hospital of Sun Yat-sen University.

### Outcome and Follow-Up

The occurrence of HF events (fatal or not) after HD was recorded. The HF diagnosis was made based on Framingham criteria (Groenewegen et al., 2020). Follow-up visits were performed by the HD centers. Patients who developed HF after HD were censored at the earliest date of a HF event. The HF events were recorded up to May 1, 2020.

### Statistical Analysis

All statistical analyses and model establishment were performed using SPSS 26.0 for Windows and Python 3.7.6. All continuous variables were described as mean ± standard deviation (SD), and these variables were compared between groups by performing t-tests. In addition, discrete variables were expressed in numbers (n) and percentages (%), and the chi-square test was applied. All results were considered to be statistically significant within a two-sided test with *p* <0.05.

Two classification models were established based on XGBoost and traditional linear logistic regression models. The multiple logistic regression was selected using a stepwise method. In the XGBoost model, all the characteristics of HD patients were included. The data set was randomly divided into a training set by 75% and a validation set by 25%. The performance of the models was evaluated using calibration curves and area under the receiver operating characteristic curves (AUCs). To evaluate the influence of different variables on the results, the SHAP value of important variables was calculated (Rodriguez-Perez and Bajorath, 2020). A risk curve was constructed using the Kaplan–Meier analysis, and differences between groups were compared by a log-rank test to further evaluate the performance of our model and verify the risk factors selected by the mode. To make our model be an easy-to-use tool, a nomogram was developed based on the risk factors selected by our model.

## Results

### Demographic and Clinical Characteristics of Study Population

According to the inclusion and exclusion criteria, a total of 353 patients were finally included in this study. The basic variables of study population are shown in Table 1. Among these patients, 96 patients (73 males and 23 females) developed HF during the follow-up duration and 257 patients (166 males and 91 females) did not. In general, the average age of these patients was 54.92 ± 15.29, and the proportion of males was 67.71%. Compared with patients without HF, those with HF were older and had a higher ratio of hypertension and diabetes, and there was a significant difference in gender. In terms of laboratory data, for patients in the HF group, their WBC, NEUT, CRP, and TIBC levels were significantly (*p* <0.05) higher than those of the other group. There was no significant difference in other characteristics.

**TABLE 1:** Clinical characteristics and laboratory parameters of patients in the HF group and the non-HF group.

Clinical characteristic and laboratory parameter	All patients	HF	Non-HF	*p*-value
Age (years)	56.05 (43.13–66.32)	63.1 (54.64–72.03)	52.0 (41.0–61.1)	<0.001
Gender (male) (n, %)	239 (66.71)	73 (76.04)	166 (64.59)	0.041
HTN (n, %)	281 (79.60)	88 (91.67)	193 (75.10)	<0.001
DM (n, %)	123 (34.84)	43 (44.79)	80 (31.13)	0.017
HB (g/L)	96.44 ± 21.01	97.71 ± 21.52	95.98 ± 20.96	0.423
NEUT (10^9^/L)	4.39 (3.47–5.60)	4.86 (3.50–6.41)	4.26 (3.44–5.17)	0.005
WBC (10^9^/L)	6.72 (5.36–8.03)	7.68 (6.07–9.00)	6.48 (5.20–7.69)	<0.001
LYMPH (10^9^/L)	1.19 (0.89–1.50)	1.18 (0.90–1.54)	1.20 (0.89–1.49)	0.854
IPTH (pg/ml)	343.40 (213.43–529.19)	322.50 (192.51–465.64)	356.45 (218.37–536.82)	0.128
UA(μmol/L)	494.88 ± 146.20	513.74 ± 154.06	487.81 ± 142.83	0.861
BUN (mmol/L)	23.07 (16.39–29.09)	23.27 (18.30–31.25)	22.85 (15.96–28.69)	0.299
CRE (μmol/L)	848.60 ± 391.62	812.26 ± 372.39	862.18 ± 398.40	0.329
CA (mmol/L)	2.17 (2.04–2.32)	2.14 (1.99–2.30)	2.19 (2.07–2.33)	0.103
P (mmol/L)	1.81 (1.44–2.25)	1.81 (1.51–2.20)	1.82 (1.44–2.26)	0.854
CHOL (mmol/L)	4.15 (3.62–4.89)	4.10 (3.57–4.97)	4.17 (3.65–4.87)	0.655
TG (mmol/L)	1.51 (1.17–1.92)	1.43 (1.11–1.90)	1.52 (1.20–1.93)	0.434
HDL-C (mmol/L)	0.97 (0.87–1.11)	0.99 (0.88–1.15)	0.96 (0.86–1.10)	0.572
LDL-C (mmol/L)	2.45 (2.00–2.96)	2.46 (2.00–3.15)	2.44 (1.99–2.94)	0.668
CRP (mg/L)	5.66 (2.65–13.65)	8.40 (4.05–16.35)	5.01 (2.12–13.00)	<0.001
TIBC (mmol/L)	36.16 (33.73–39.55)	35.84 (33.48–38.09)	36.46 (33.95–40.00)	0.034
PLT (10^9^/L)	201.00 (156.00–243.70)	210.00 (155.50–273.00)	198.72 (157.25–237.00)	0.177

CRP, C-reactive protein; BUN, blood urea nitrogen; CA, calcium; HB, hemoglobin; DM, diabetes mellitus; TG, triglycerides; P, phosphorus; CHOL, cholesterol; CRE, serum creatinine; LYMPH, lymphocyte; UA, uric acid; LDL-C, low-density lipoprotein cholesterol; HDL-C, high-density lipoprotein cholesterol; IPTH, intact parathyroid hormone; WBC, white blood cell count; NEUT, neutrophil count; PLT, platelet count; TIBC, total iron-binding capacity; HTN, hypertension.

### Logistic Regression Analysis

**FIGURE 1:**
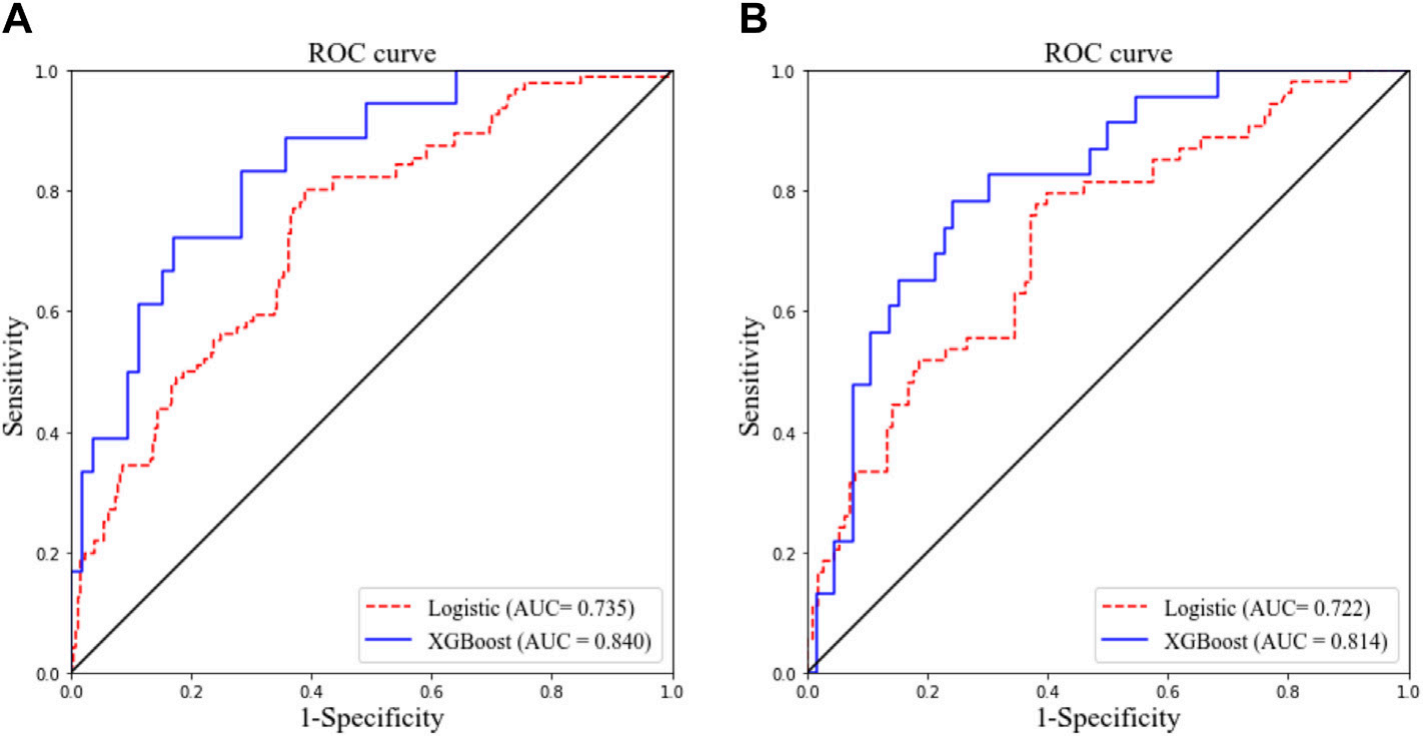
Receiver operating characteristic curves of two models for predicting objective response in the training cohort **(A)** and validation cohort **(B)**. AUC, area under the curve.

**FIGURE 2:**
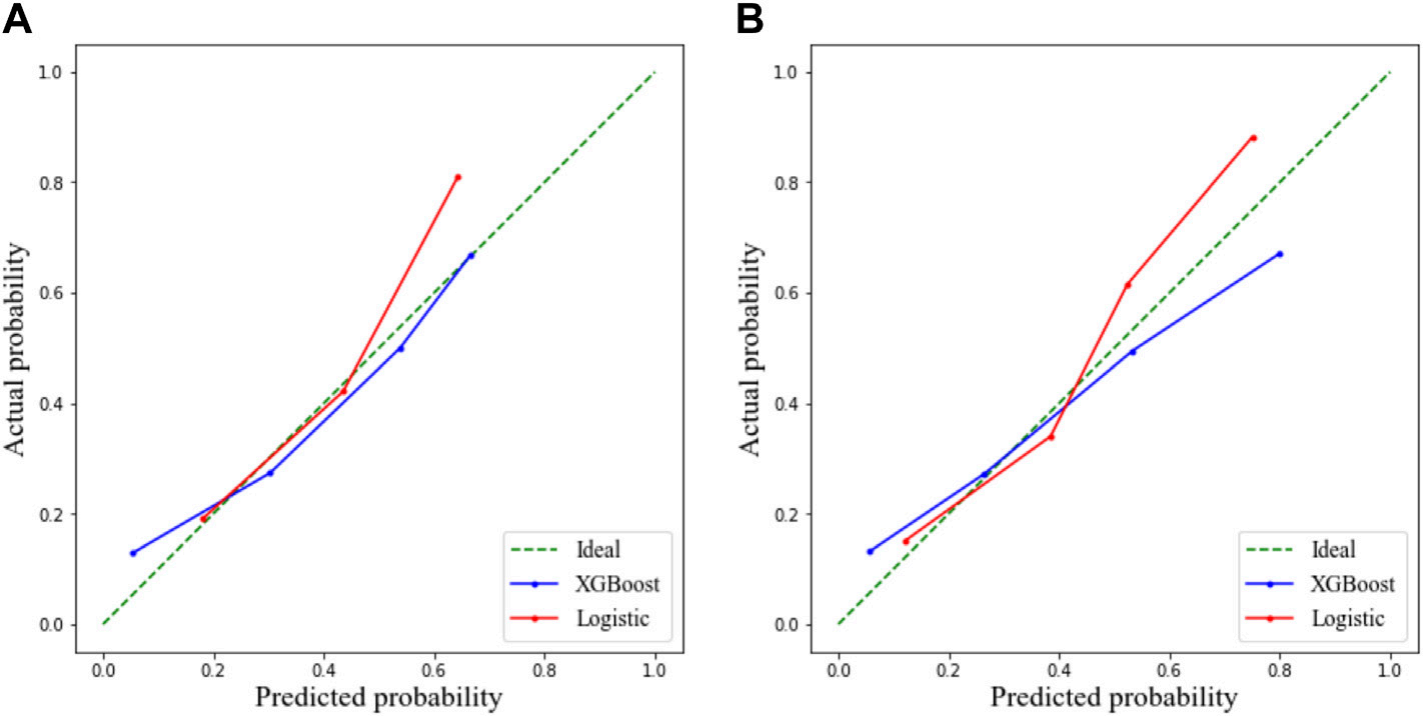
Calibration curve demonstrating predictions from the model to the actual observed probability. **(A)** Training cohort and **(B)** validation cohort.

As presented in Table 2, the following factors were included in a multivariate stepwise logistic regression model: HTN (OR: 3.786, 95% CI: 1.640–8.739, and *p* = 0.002), WBC (OR: 1.115, 95% CI: 1.026–1.212, and *p* = 0.011), CRP (OR: 1.020, 95% CI: 1.009–1.032, and *p* = 0.001), and age (OR: 1.026, 95% CI: 1.008–1.044, and *p* = 0.004). The receiver operating characteristic (ROC) curve of the logistic regression model is shown in Figure 1, and the performance of this model was evaluated using the calibration curve, as shown in Figure 2. The area under the curve (AUC) was 0.722 for the validation group and 0.735 for the training group. In addition, accuracy, sensitivity, and specificity of the model were 74.8, 75.6, and 74.4%, respectively, evaluated by the validation group.

**TABLE 2:** Multivariate logistic regression model.

Variable	B	SE	Wald	*p*-value	OR (95%CI)
HTN	1.331	0.427	9.733	0.002	3.786 (1.640–8.739)
WBC	0.109	0.043	6.532	0.011	1.115 (1.026–1.212)
CRP	0.020	0.006	11.730	0.001	1.020 (1.009–1.032)
AGE	0.025	0.009	8.113	0.004	1.026 (1.008–1.044)

HTN, hypertension; WBC, white blood cell count; CRP, C-reactive protein; OR, odds ratio; CI, confidence interval.

### Extreme Gradient Boosting Model

**FIGURE 3:**
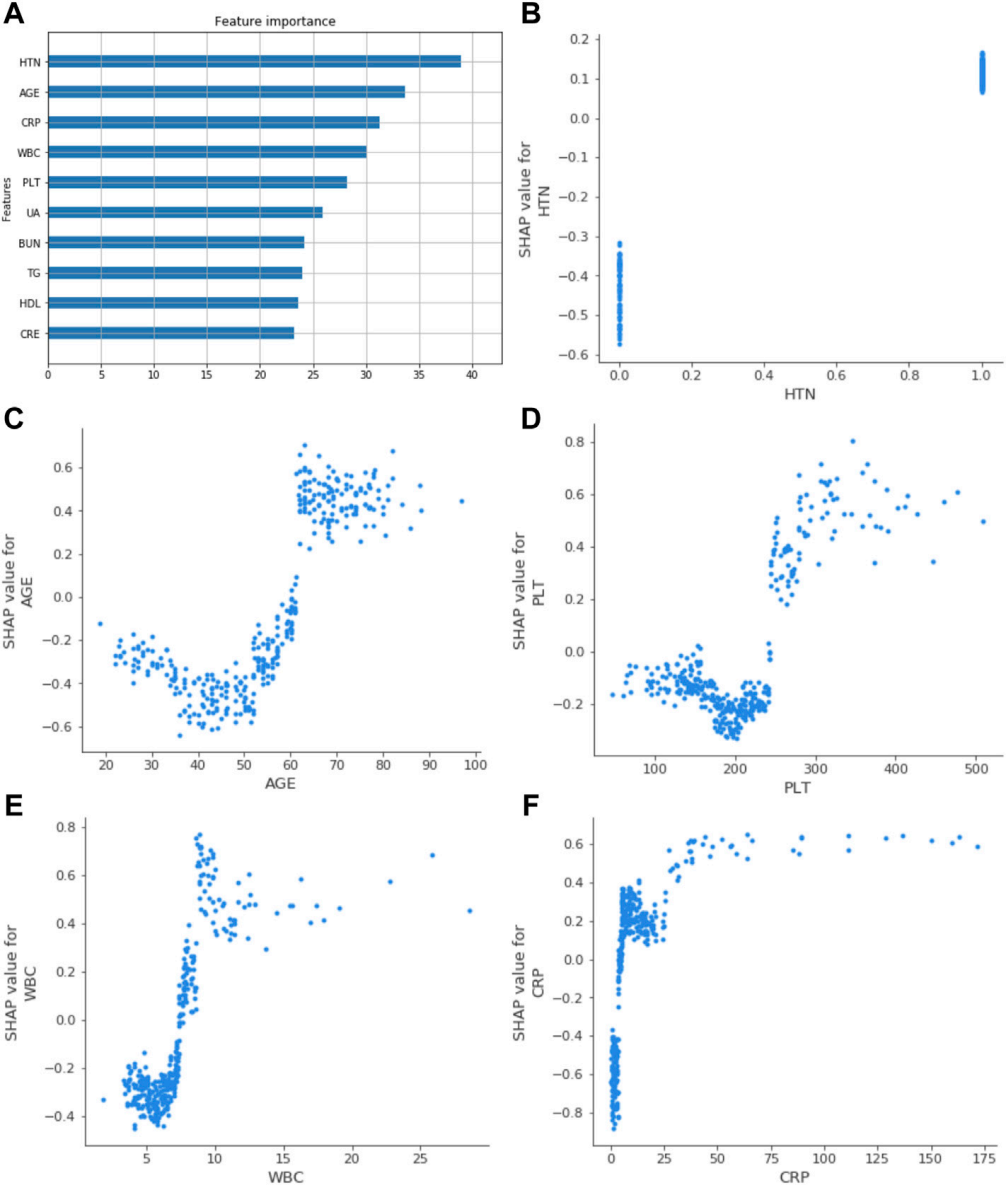
Related factors of HF were ranked by feature gain. **(A)** showed the top ten gain features; age, HTN, WBC, PLT, and CRP had a more significant impact. **(B)**, **(C)**, **(D)**, **(E)**, and **(F)** represented the SHAP value of HTN, age, PLT, WBC, and CRP in the XGBoost model. It reflected the influence of relevant factors on the results. WBC, white blood cell count; HTN, hypertension; UA, uric acid; TG, triglycerides; BUN, blood urea nitrogen; HDL, high-density lipoprotein cholesterol; PLT, platelet count; CRE, serum creatinine; CRP, C-reactive protein.

The ROC curve and the calibration curve of the XGBoost model are presented in Figure 1 and Figure 2, respectively. It was found that the accuracy, sensitivity, specificity, and AUC value were 78.5, 79.6, 78.1%, and 0.814, respectively, evaluated by the validation cohort. The feature importance ranking which represents the contribution of the corresponding feature to the model prediction based on gain is shown in Figure 3. It was found that HTN, age, CRP, WBC, and PLT were more important for the prediction of HC than other features.

### Interpretation of the Extreme Gradient Boosting Model

The SHAP value quantifies the contribution of data to the outcome in the XGBoost model; the size of the value reflects the influence of the corresponding eigenvalue on the outcome, and the positive and negative values reflect whether the results are promoted. Based on the established XGBoost model, the SHAP values of the five relevant factors showed how the feature affected the results, as shown in Figure 3.

### Kaplan–Meier Analysis

**FIGURE 4:**
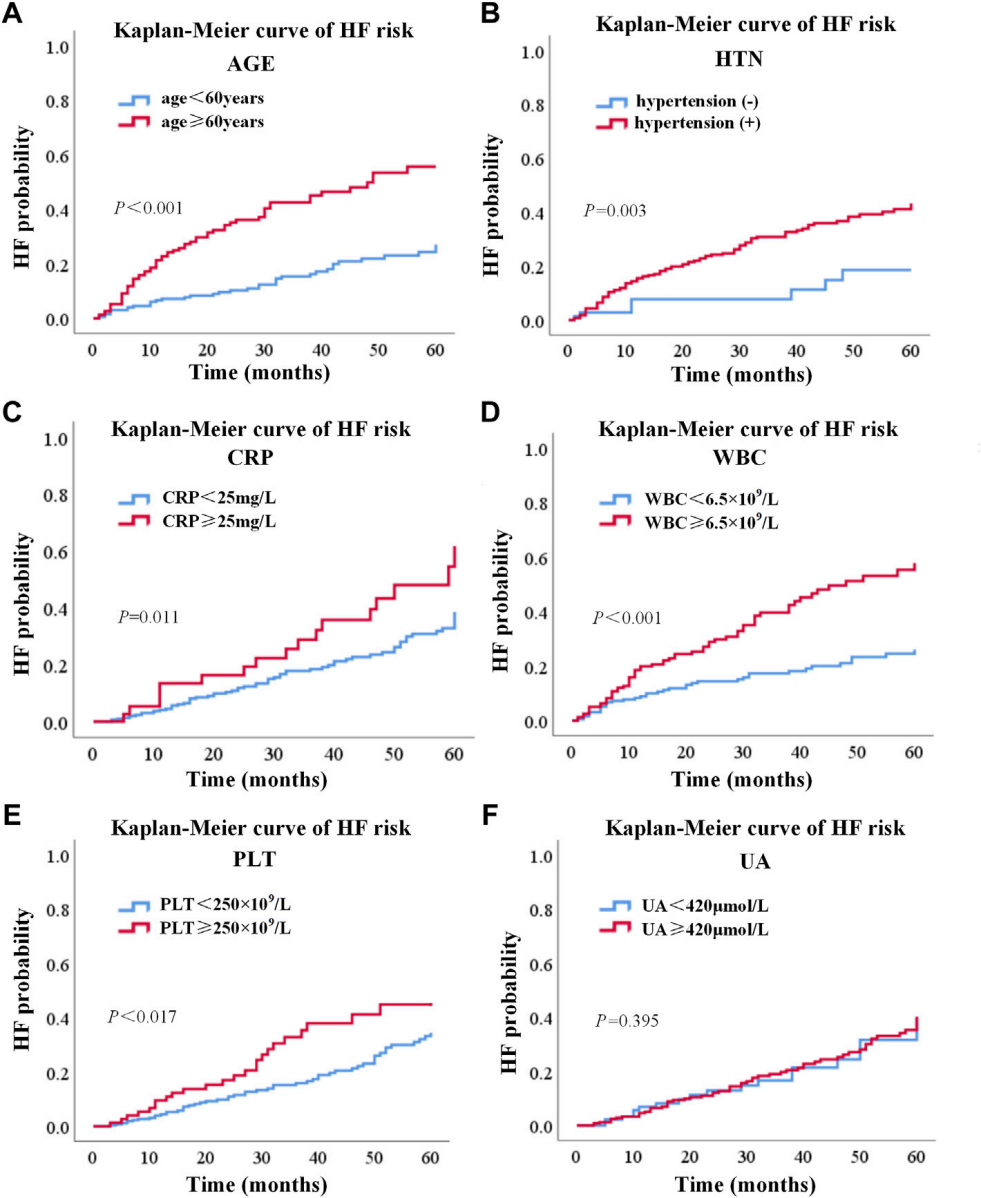
Risk curve of six factors based on the Kaplan–Meier analysis. **(A)**, **(B)**, **(C)**, **(D)**, **(E)**, and **(F)** represented risk curves of age, hypertension, CRP, WBC, PLT, and UA, respectively. WBC, white blood cell count; UA, uric acid; CRP, C-reactive protein; PLT, platelet count.

**FIGURE 5:**
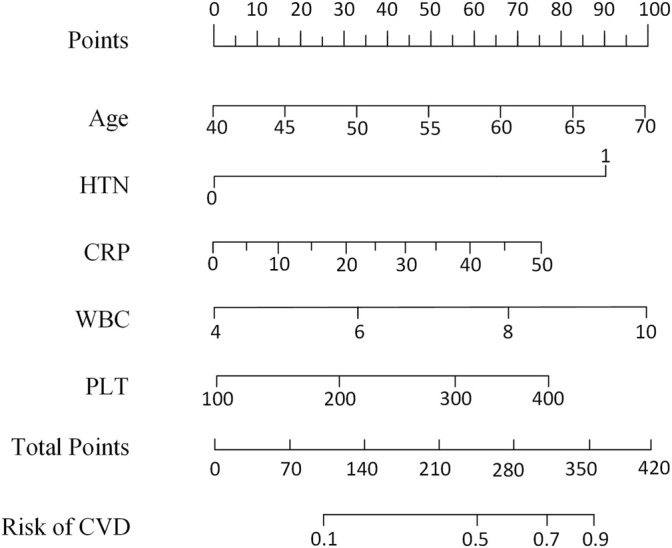
Nomogram scaled by the coefficient of each risk factor selected by the XGBoost model. HTN, hypertension; WBC, white blood cell count; CVD, cardiovascular disease; CRP, C-reactive protein; PLT, platelet count.

The Kaplan–Meier analysis was used to confirm the effects of risk factors on the incidence of HF. The risk factors were stratified by the SHAP value when it was 0. As shown in Figure 4, the risk curve revealed that considering the influence of different outcome time, patients with high levels of WBC, CRP, PLT, old age, and hypertension had a higher incidence of HF, and the effect of UA was not obvious enough. The nomogram of the XGBoost model was constructed, as shown in Figure 5.

## Discussion

In this retrospective study, we established and validated a clinical prediction model to predict HF in HD patients based on the XGBoost algorithm. The results indicated that our model could effectively identify the individuals who suffer from HF using routinely clinical parameters. Our result indicated that XGBoost as a non-linear model had better performance than the logistic model and showed stronger ability in identification of risk factors.

In recent years, machine learning has been widely used in risk prediction and disease screening, which has obtained excellent performance (Kwon et al., 2020; Meng et al., 2020; Cobb et al., 2021; Koteluk et al., 2021; Kourou et al., 2021). Therefore, in this research, XGBoost, an integrated machine learning algorithm, was applied to identify the complex non-linear relationship between HF and clinical variables, as well as to evaluate the importance of the variables to the HF. Although traditional multivariable analysis methods have been applied for the identification of HF in HD patients (Gedfew et al., 2020; Bramania et al., 2021), to our knowledge, this was the first machine learning model for the prediction of HF. Our results showed that the performance of the XGBoost model was better than the traditional logistic regression model in prediction of HF. To make our XGBoost model interpretable, the feature importance and SHAP value were applied to evaluate the contribution of variables to HF. Except four risk factors including age, hypertension, WBC, and CRP found in the logistic regression model, a new risk factor, PLT, was found in the XGBoost model. This showed the advantages of our XGBoost mode, which could improve the accuracy of the classification and the efficiency of identification.

In addition to HF response, the performance of our model was evaluated by the Kaplan–Meier analysis. The differences between the Kaplan–Meier curves of different risk levels indicated that the risk factors selected by our XGBoost model were important prognostic factors of HF. The results of Kaplan–Meier analysis also showed that age more than 60, WBC more than 6.5 × 10^9^, CRP more than 15 mg/L, PLT more than 250 × 10^9^, and hypertension were independent risk factors which could increase the probability of HF. Moreover, based on the five risk factors selected by the XGBoost model and the Kaplan–Meier analysis, the nomogram (Yao et al., 2019) was designed to give an easy-to-use tool for the prediction of HF.

In our study, the proportion of hypertension in HF patients was significantly greater than that in the non-HF group, which is consistent with a recent study (Bramania et al., 2021). The analysis of characteristics showed that hypertension was a risk factor for HF events, which is consistent with the results of a recent study (Cozzolino et al., 2017). Patients with hypertension already had a huge cardiovascular burden before dialysis (Van Buren, 2016). There are pieces of evidence that dialysis treatment can lead to abnormal fluctuations in blood pressure and affect patient’s hemodynamics (Chou et al., 2018; Douvris et al., 2019). The vascular intima is more damaged by the abnormal fluctuations in the blood pressure, and HF is more likely to occur (Buren and Inrig, 2012).

Age is an important risk factor of HF. In old age, the shape and function of the vascular wall are changed due to oxidative stress, cell aging, and inflammation (Ghebre et al., 2016). In addition, with increasing age, body functions gradually decline, physiological compensatory function decreases, chronic diseases such as high blood pressure and diabetes occur frequently, and symptoms such as anemia, malnutrition, and chronic inflammation are prone to occur; these factors increase the risk of HF events (McHugh and Gil, 2018; Groenewegen et al., 2020). It has been reported that approximately 80% of HF patients were more than 60 years old, and the prevalence increased with age (Vigen et al., 2012). According to the results of our study, the risk of HF continued to increase from about 45 years old, which was consistent with the report from the American Heart Association (Virani et al., 2021).

WBC and CRP are widely used to reflect the inflammatory state in patients (Rienstra et al., 2012; Adamo et al., 2020). Inflammation and HF are strongly interconnected, and higher levels of inflammation markers indicated an increased mortality and morbidity of HF in patients (Ekdahl et al., 2017). Some studies have confirmed that inflammation markers are implicated in the development of HF because the immune system leads to deterioration of the structure and function of the cardiovascular system by the inflammatory response (Van Linthout and Tschope, 2017; Castillo et al., 2020). A study has demonstrated that high levels of WBC are closely related to the incidence of HF (Bekwelem et al., 2011). CRP is one of the recognized risk factors for CVD events, and a meta-analysis has reported that CRP plays an important role in the development of HF (Emerging Risk Factors Collaboration et al., 2010). In this study, our result was consistent with these previous findings. In addition, our results showed that other serum biomarkers, such as an anomalous level of PLT, were related to HF. Excessive PLT can promote inflammation, negatively affect the left ventricular function, and increase the risk of atherosclerosis; these diseases would increase the probability of HF (Gary et al., 2013; Chen and Yang, 2020).

All these data required in this study can be easily obtained through routine clinical examination, not limited by many additional conditions, and can make full use of existing resources. The XGBoost model showed a better prediction effect by using higher indicators such as AUC, accuracy, sensitivity, and atherosclerosis specificity. It can be a useful tool for doctors to evaluate the HF risk of HD patients and carry out personalized intervention in advance. However, there are still some limitations to our study. We only focused on the event of HF in HD population of China; it needs to be verified by different populations and races. In addition, this study is a retrospective examination which lacks longitudinal observation. Therefore, in further research, a longitudinal data analysis can help evaluate the changes in these risk factors over time and further validate the results in our model.

In conclusion, we developed and validated a clinical prediction model based on the XGBoost algorithm for HF in end-stage renal disease patients. The model had an excellent predictive performance and provided a useful tool for the early prediction of HF in HD patients.

## Data Availability

The raw data supporting the conclusions of this article will be made available by the authors, without undue reservation.
